# Genetic mutation and blue rubber bleb nevus syndrome: case reports and literature review

**DOI:** 10.3389/fgene.2025.1516562

**Published:** 2025-06-13

**Authors:** Yueyi Xing, Han Liu, Hua Liu, Xueli Ding, Xue Jing

**Affiliations:** ^1^ Medical College, Qingdao University, Qingdao, Shandong, China; ^2^ Gastroenterology Department, The First Affiliated Hospital of the University of South China, Hengyang, Hunan, China; ^3^ Gastroenterology Department, The Affiliated Hospital of Qingdao University, Qingdao, Shandong, China

**Keywords:** blue rubber bleb nevus syndrome, genetic mutation, TEK, venous malformations, gastrointestinal hemorrhage

## Abstract

Blue Rubber Bleb Nevus Syndrome (BRBNS) (OMIM %112200), or Bean syndrome, is an infrequent disorder characterized by venous malformations (VaMs) involving various organs such as the skin and gastrointestinal tract. Genetic mutations that affect the proliferation, migration, adhesion, differentiation, and survival of endothelial cells and the integrity of the extracellular matrix may be the pathogenesis of these disorders. We are supposed to investigate the cytogenetic results of BRBNS and report two sporadic cases. Two unrelated cases with BRBNS were from the Affiliated Hospital of Qingdao University and the First Affiliated Hospital of the University of South China, respectively. The data collection included information on the current age, sex, and race of the individuals, as well as their chief complaint. Clinical and family history, physical and laboratory findings, diagnostic workup, results, treatment, and complications were all documented. We are supposed to investigate the cytogenetic results of BRBNS and report two sporadic cases. We identified TEK missense mutations (c.596A>C) in both participants with BRBN. In addition, the mutation has appeared in *MMP9, NOTCH3, PRSS1, PDGFRA, CCM2, TSC2*, and *TNFAIP6*. KEGG pathway analysis showed that they participated in the PI3K-AKT signaling pathway. Our findings underscore the importance of exploring these genetic alterations in the context of BRBNS, which may have implications for developing targeted therapeutic approaches. We present two cases diagnosed with Bean syndrome, detailing their clinical features and molecular aspects.

## 1 Introduction

Blue rubber bleb nevus syndrome (BRBNS) (OMIM %112200), also known as Bean Syndrome, is a rare genetic disorder described by Gascoyne in 1860. However, it was not fully defined until 1958 by William Bean; therefore, it became known as “Bean syndrome” (Bean, 1957). Propositus presents with multiple venous malformations in various organ systems, including the liver, spleen, heart, eye, and central nervous system. Propositus have dozens to hundreds of lesions, which usually increase with age in size and number (Oranje, 1986). Propositus with blue rubber bleb nevus syndrome are at increased risk for gastrointestinal hemorrhage and severe iron deficiency anemia. It may also have serious complications involving rupture, intestinal volvulus, and intussusception, and may even lead to death. Some studies have shown that venous malformations are related to *TIE2* gene mutations (Vikkula et al., 1996; Limaye et al., 2009).

BRBNS is usually caused by sporadic mutations in the TEK gene encoding the TIE2-angiopoietin endothelial tyrosine kinase receptor (Soblet et al., 2017; Mayba and Cullingham, 2019), but only a few germline mutations are located on chromosome 9p (Gallione et al., 1995; Wang et al., 2018). A study showed that rare *GLMN* germline variants may contribute to BRBN (Yin et al., 2019). Genetic sequencing was performed on both cases to gain insight into the pathogenesis of BRBNS and determine the underlying genetic factors that may contribute to the manifestation of BRBNS. The results obtained from this study may provide valuable information for better understanding and managing this condition.

## 2 Materials and methods

This report details two BRBNS cases. One case was from the Affiliated Hospital of Qingdao University, and the other was from the First Affiliated Hospital of the University of South China. The report includes each case’s medical history, symptoms, and treatments. This report aims to contribute to the medical community’s understanding of BRBNS. The diagnosis of BRBNS was conclusively determined through endoscopy and pathology. Data on current age, sex, race, chief complaint, clinical and family history, physical and laboratory findings, diagnostic workup and results, treatment, and complications were collected. Blood samples were used to prepare genomic DNA. The method used for detecting Whole Exome Sequencing (WES) was Next-Generation Sequencing (NGS) technology provided by BGI Genomics Co., Ltd. (Shenzhen). The Ethics Committee of the Affiliated Hospital of Qingdao University (QYFY WZLL 28596) and The First Affiliated Hospital of the University of South China (2024LL0325001) have approved the DNA test.

GeneMANIA (http://genemania.org/) and STRING (https://string-db.org/) explored gene interactions and functions and identified co-expression, co-localization, and shared protein domain genes. GO and KEGG enrichment analyses were performed using R software.

## 3 Results

### 3.1 Case reports

**FIGURE 1:**
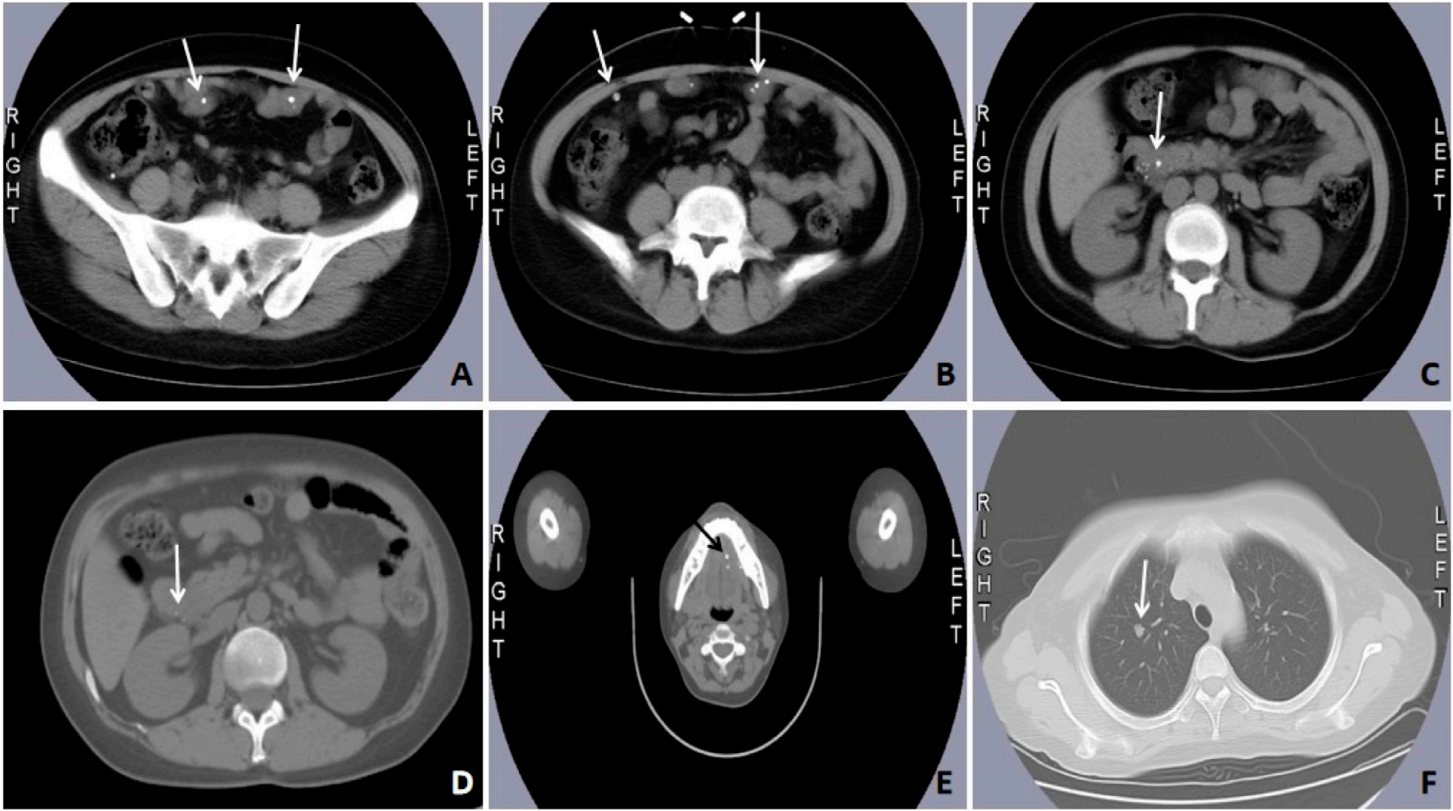
Computed tomography imaging and the positron emission tomography/computed tomography (PET/CT) scan of case #1. Abdominal CT showed multiple spotted calcifications in the intestine **(A–C)** and the head of the pancreas **(D)**. Tomography/computed tomography (PET/CT) scan showed multiple spotted calcifications in the hard palate **(E)**. Chest CT showed a soft tissue mass shadow in the lung **(F)** (marked by the white or black arrows).

**FIGURE 2:**
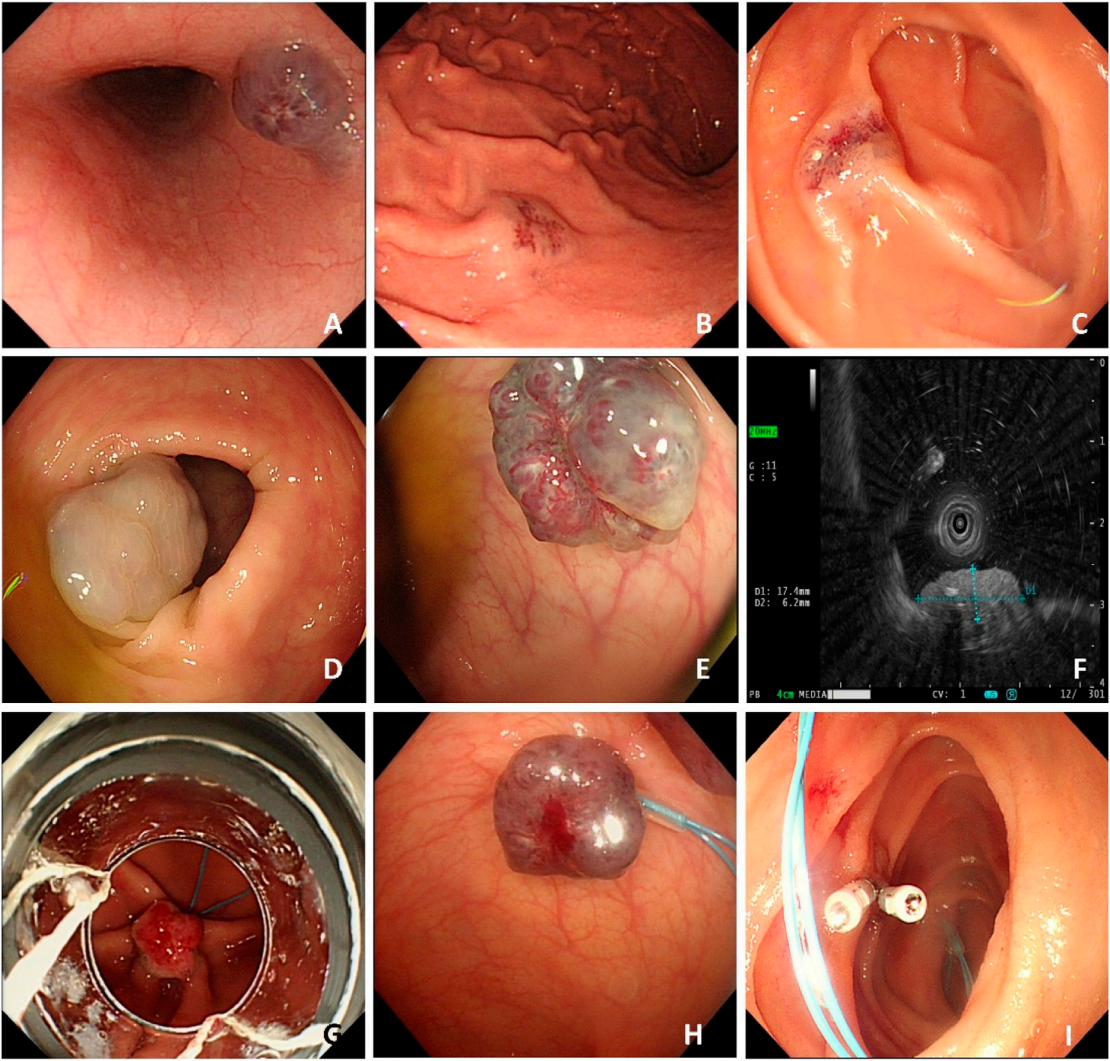
Case#1 has multiple gastrointestinal vascular malformations observed during gastrointestinal endoscopy. Multiple venous malformations were detected in the esophagus **(A)**, gastric body **(B)**, intestine **(C)**, and colon **(D,E)**. Endoscopic ultra-sonography (EUS) shows a hyperechoic lesion with a distinct anterior border originating from the submucosal layer **(F)**. This case received an endoscopic ligation and titanium clips **(G–I)** placement.

Case #1, a 32-year-old Chinese female, was admitted in November 2017, firstly due to a history of persistent fatigue spanning over 20 years. The case had previously been diagnosed with iron deficiency anemia (IDA), and oral ferrous fumarate was prescribed to treat fatigue. Colds and post-menstrual periods exacerbated the fatigue episodes. Further investigations included bone marrow aspiration, which did not reveal any evidence of malignant clonal disease within the hematopoietic system. Thus, the case was visited by the Affiliated Hospital of Qingdao University for further diagnosis and treatment. She has had ovarian cysts for 2 years but denied having melena, hematochezia, recurrent epistaxis, hemoptysis, hematemesis, and stomachache. Due to family upheavals, it was impossible to trace other family members, making it impossible to rule out a family history completely. Physical examination showed multiple blue rashes on hands, feet, tongue, and back, which are higher than the skin surface, clearly defined, not easy to push, without bleeding ulcer on the surface, incomplete fading after pressing, non-itchy, and non-painful. Laboratory results showed severe microcytic hypochromic anemia and a weakly positive fecal occult blood test (FOBT). Computed tomography (CT) and the positron emission tomography/computed tomography (PET/CT) scan indicated multiple nodules in the descending duodenum, small intestine, colon, lung, lateral genioglossus, hard palate, right shoulder and back, as well as the left side of the chest (Figure 1). Gastrointestinal and capsule endoscopy showed multiple hemangiomas in the esophagus, stomach, small intestine, and colon (Figure 2). Histopathology of left abdominal hemangioma resection biopsy confirmed the diagnosis of Blue Rubber Bleb Nevus Syndrome (BRBNS). The case underwent treatment with endoscopic ligation and oral sirolimus but continued to experience fatigue and hematochezia post-discharge. Subsequent blood tests revealed hemoglobin 70 g/L, necessitating repeat endoscopic ligation. Upon further management and follow-up, it is evident that the symptoms have shown improvement.

**FIGURE 3:**
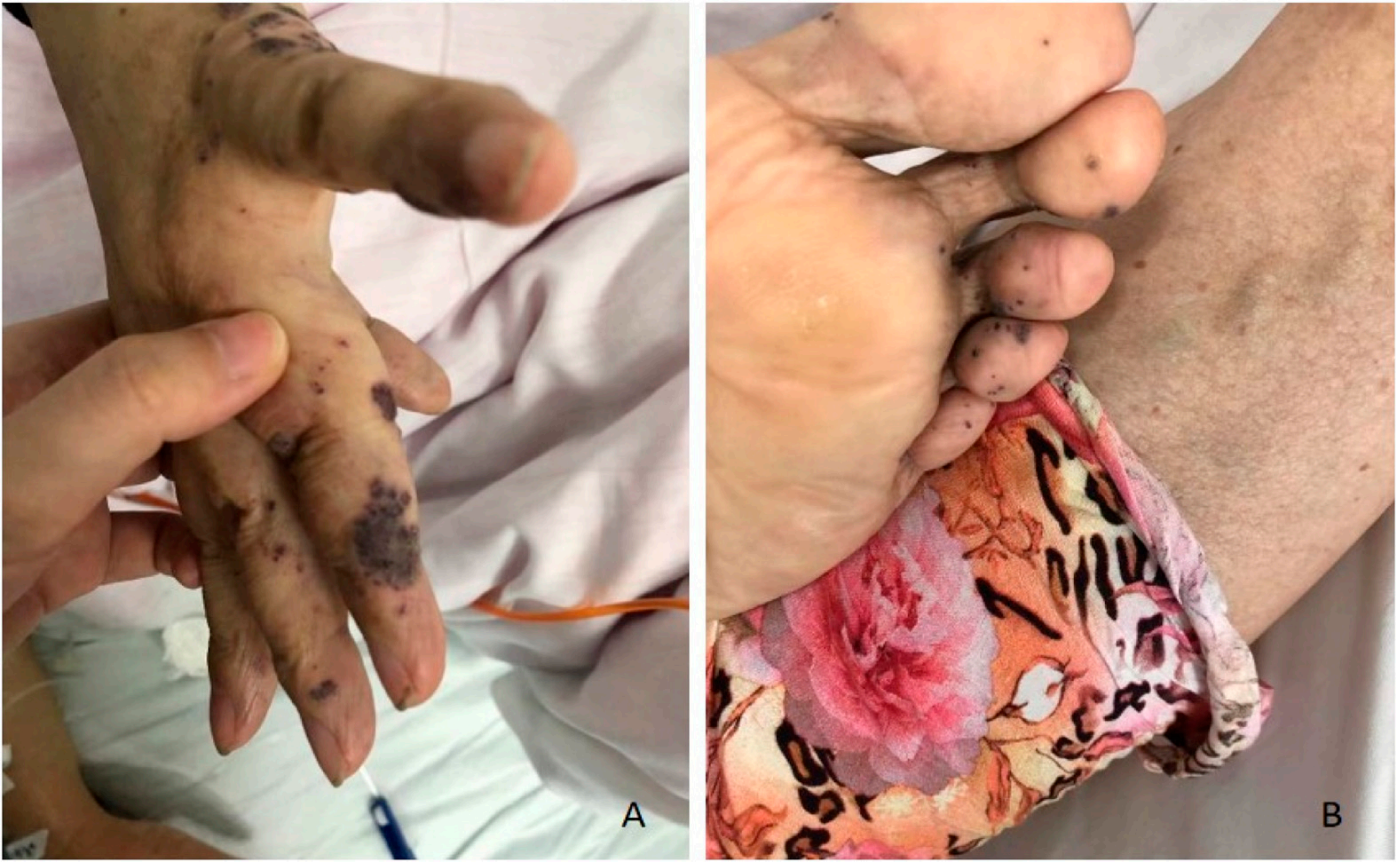
Representative images of skin lesions of case #2. Several soft, blue, rubbery nodules are on the hands **(A)** and feet **(B)**.

**FIGURE 4:**
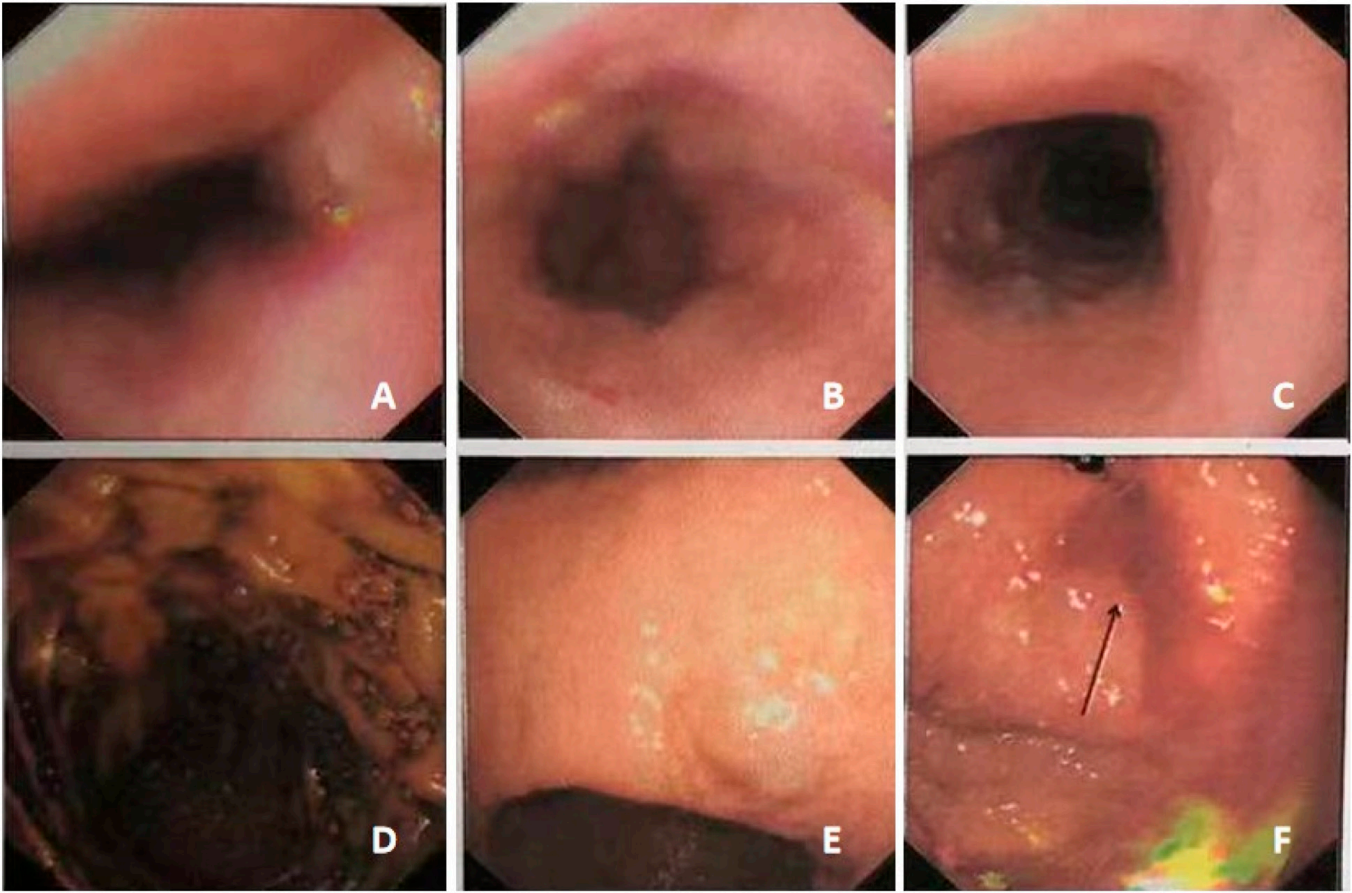
Gastroscopy showed multiple gastrointestinal vascular malformations in case #2. The middle and lower segments of the esophagus showed multiple hemangiomas **(A–C)**. The gastric body showed multiple bluish-purple hemangiomas **(D–F)**.

Case #2, a 57-year-old Chinese female, was admitted to the hospital in July 2020 due to symptoms of melena, dizziness, and fatigue persisting for 1 week. Upon admission to the local hospital, she was diagnosed with bleeding esophageal varices after undergoing a gastroscopy. Subsequently, the case sought further evaluation and treatment at The First Affiliated Hospital of the University of South China. The case had a medical history significant for viral hepatitis B; however, she denied experiencing hematochezia or recurrent episodes of epistaxis, hemoptysis, hematemesis, or stomachache. The parents of this case have both passed away, so we are unable to trace their family history. Upon physical examination, the case exhibited sporadic, bluish, rubber-like hemangiomas on her distal limbs (Figure 3). Laboratory results showed severe microcytic hypochromic anemia and a weakly positive FOBT. Upon further examination, multiple hemangiomas were found in the esophagus’ middle and lower segments and gastric body (Figure 4). The case received supportive therapy, which included oral iron supplementation and drug hemostasis. As a result, the hemoglobin levels have increased compared to previous measurements. Subsequent follow-up assessments revealed that the case’s hemoglobin remained stable.

### 3.2 Gene detection

Genes were measured and analyzed in two unrelated sporadic cases with BRBNS. As shown in Table 1, we found their key gene mutations. We identified *TEK* missense mutations (c.596A>C) in both participants with BRBN. In addition, case #2 has a missense mutation in *TEK* (c.1357G>T and c.131T>C). Apart from that, we also found missense mutation in *MMP9* (c.836A>G and c.1721G>C), *NOTCH3* (c.6668C>T), *PRSS1* (c.47C>T and c.161A>G), *PDGFRA* (c.1432T>C) and *TSC2* (c.856A>G).

**TABLE 1:** Key gene mutations of two unrelated sporadic cases with BRBNS.

Case	Gene	Genotype	Variant classification	cDNA change
#1	*TEK*	Heterozygote mutation	Missense	c.596A>C
	*MMP9*	Compound Heterozygote mutation	Missense	c.836A>G c.1721G>C
	*NOTCH3*	Heterozygote	Missense	c.6668C>T
	*PRSS1*	Compound mutation Heterozygote	Missense	c.47C>T c.161A>G
#2	*TEK*	Triallelic Heterozygote mutation	Missense	c.596A>C c.131T>C c.1357G>T
	*PDGFRA*	Heterozygote mutation	Missense	c.1432T>C
	*CCM2*	Heterozygote mutation	Splice Site	c.915G>A
	*TSC2*	Heterozygote mutation	Missense	c.856A>G
	*TNFAIP6*	Heterozygote mutation	Delete Stop Codon	-

Heterozygote mutation: Two alleles of a gene have different mutations. Compound Heterozygote mutation: Abnormal mutations at two distinct genetic loci co-occur on the same chromosome in the same individual. Triallelic Heterozygote mutation: Three allelic variants coexist at a single genetic locus.

**FIGURE 5:**
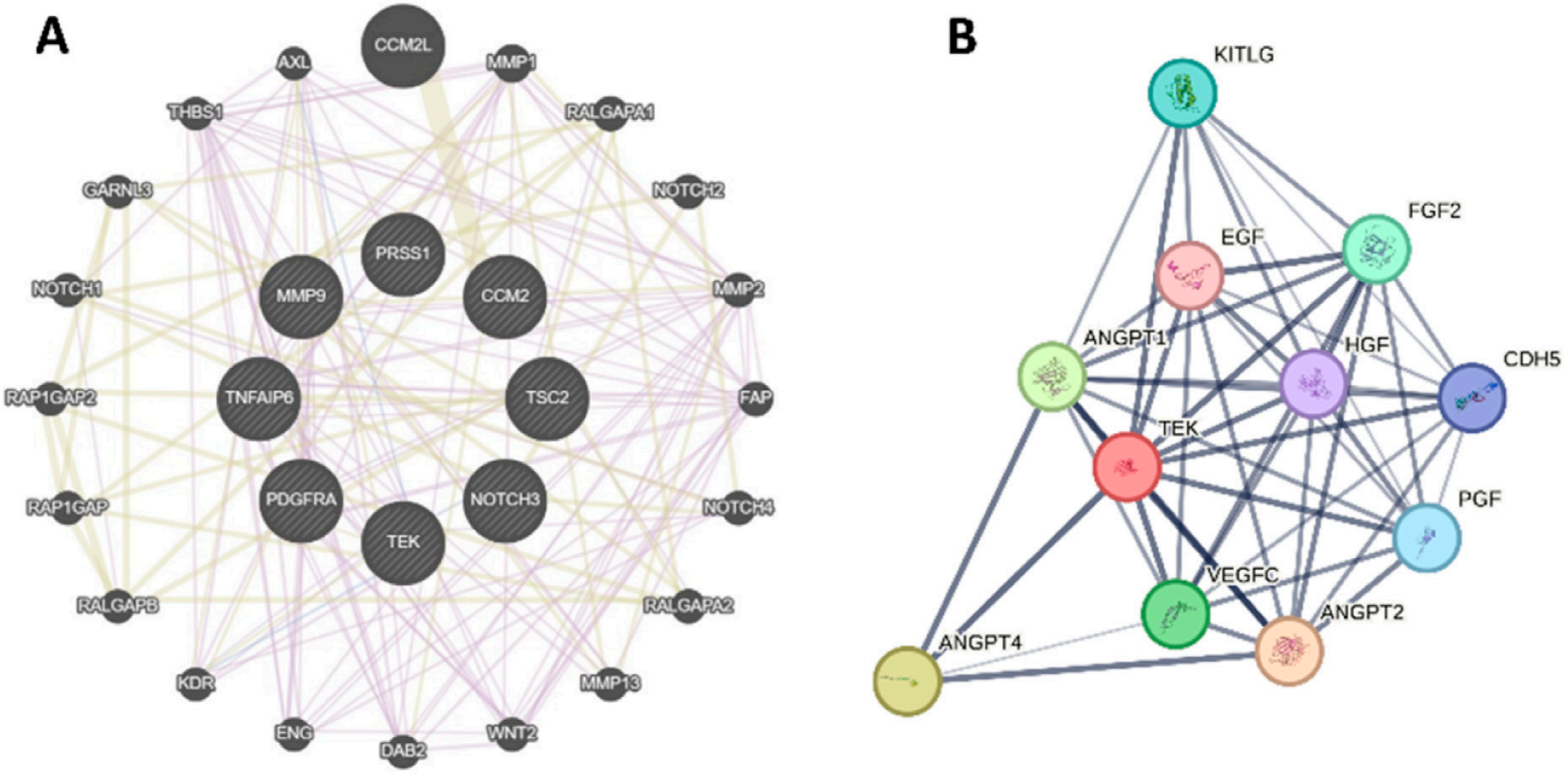
Protein-protein interaction (PPI) network. **(A)** The PPI of *TEK, MMP9, NOTCH3, PRSS1, PDGFRA, CCM2, TSC2,* and *TNFAIP6*. **(B)** The PPI of TEK-related genes.

**FIGURE 6:**
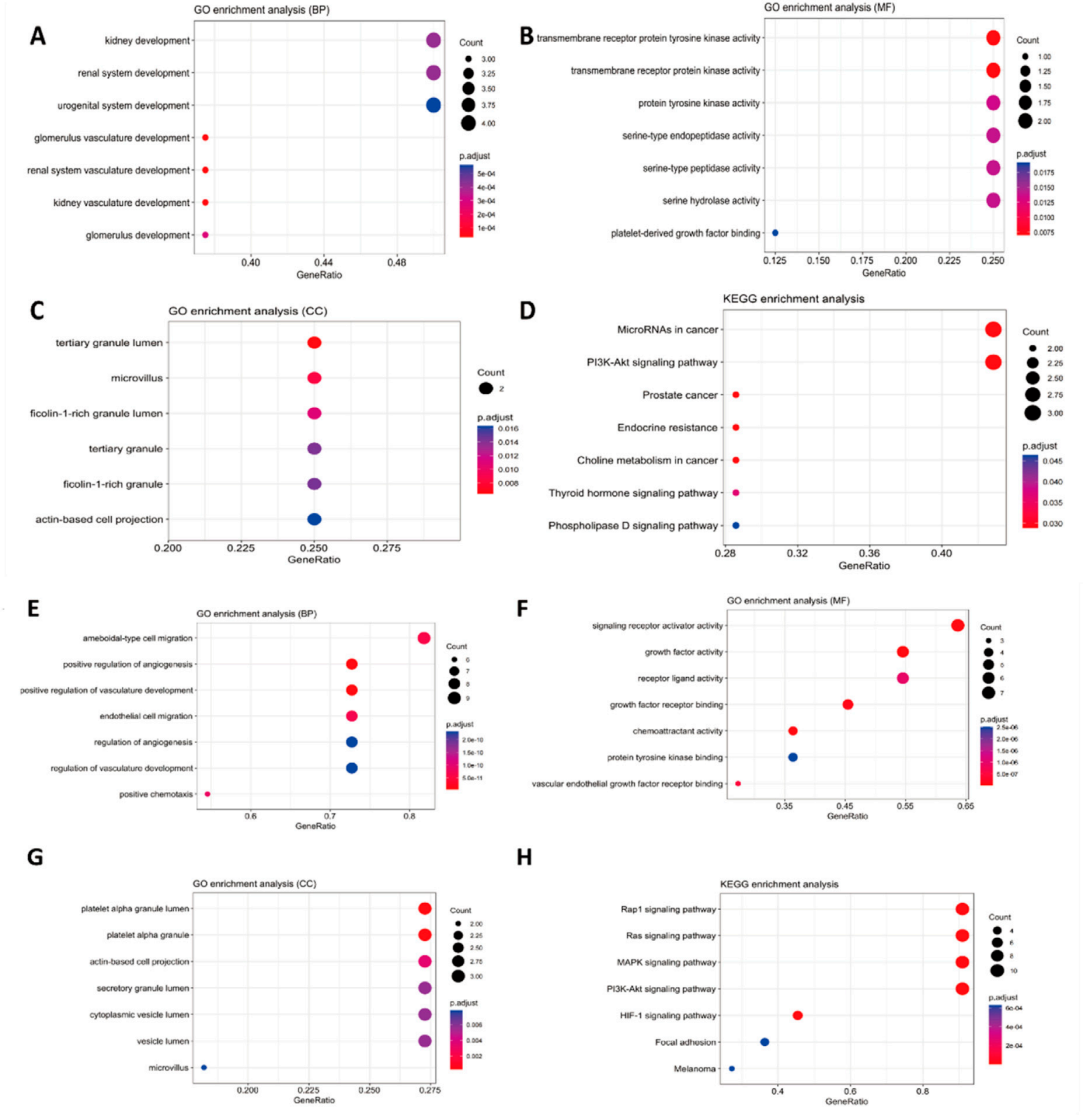
GO and KEGG Enrichment analysis. **(A–D)** BP enrichment analysis, MF enrichment analysis, CC enrichment analysis, and KEGG enrichment analysis of *TEK, MMP9, NOTCH3, PRSS1, PDGFRA, CCM2, TSC2*, and *TNFAIP6*. **(E–H)** BP enrichment analysis, MF enrichment analysis, CC enrichment analysis, and KEGG enrichment analysis of *TEK*-related genes.

To further study the molecular mechanism of these genes, we performed a protein-protein interaction (PPI) network of *TEK, MMP9, NOTCH3, PRSS1, PDGFRA, CCM2, TSC2,* and *TNFAIP6* (Figure 5A), and *TEK*-related genes (Figure 5B). Genomic functional annotation was conducted through Gene Ontology (GO) and KEGG pathway enrichment analyses of pivotal genes. Biological process (BP) categorization revealed predominant associations with nephrogenesis, including kidney development, renal system development, urogenital system development, glomerulus vasculature development, renal system vasculature development, kidney vasculature development, and glomerulus development (Figure 6A). Molecular function (MF) characterization demonstrated significant enrichment in enzymatic activities, particularly transmembrane receptor protein tyrosine kinase activity, transmembrane receptor protein kinase activity, protein tyrosine kinase activity, serine-type endopeptidase activity, serine-type peptidase activity, serine hydrolase activity, and platelet-derived growth factor binding (Figure 6B). Cellular component (CC) analysis identified prominent localization within specialized subcellular structures, including tertiary granule lumen, microvillus, ficolin-1-rich granule lumen, tertiary granule, ficolin-1-rich granule, and actin-based cell projection (Figure 6C). KEGG pathway mapping indicated participation in eight oncogenic and metabolic pathways, particularly microRNA-related carcinogenesis, PI3K-AKT signaling pathway, Prostate cancer, Endocrine resistance, Choline metabolism in cancer, Thyroid hormone signaling pathway, Phospholipase D signaling pathway (Figure 6D). Distinct functional patterns emerged from TEK-associated gene profiling. BP analysis of TEK-related elements highlighted regulatory roles in vascular morphogenic processes, including angiogenic modulation, endothelial motility, and chemotropic responses (Figure 6E). MF assessment revealed critical involvement in signal transduction mechanisms, particularly growth factor receptor interactions, chemotactic signaling, and tyrosine kinase binding capacities (Figure 6F). CC distribution analysis demonstrated compartmentalization within secretory vesicular structures, including platelet α-granule matrices and cytoplasmic vesicular lumina (Figure 6G). KEGG pathway integration analysis implicated TEK-related genes in multiple signaling networks, particularly Rap1/Ras/MAPK cascades, HIF-1-mediated hypoxia responses, and focal adhesion complexes (Figure 6H).

## 4 Discussion

BRBNS is a rare genetic disorder that can manifest at any age and in any sex. The syndrome affects people of all races, but it is most common in White individuals (Zahedi et al., 2013). While cutaneous symptoms typically appear at birth or early childhood, there have been reports of diagnosis as late as 89 (Aron et al., 2018). The incidence of BRBNS is generally considered a sporadic disorder, occurring in about 1 in every 14,000 births (Martinez et al., 2014). Individuals diagnosed with blue rubber bleb nevus syndrome have a higher likelihood of experiencing gastrointestinal bleeding and developing severe anemia due to iron deficiency (Nahm et al., 2004). These symptoms were also present in both of our cases.

BRBNS is a sporadic disorder, but there have been reports of autosomal dominant inheritance, particularly with a locus identified on chromosome 9p (Dòmini et al., 2002). Recently, somatic mutations in *TIE2*, a receptor for angiopoietins on endothelial cells, have been discovered. Sublet et al. have identified somatic mutations in the *TEK* gene, which encodes *TIE2*, a tyrosine kinase receptor involved in various stages of angiogenesis, as the primary cause of BRBNS (Soblet et al., 2017). These stages include destabilizing existing blood vessels, guiding endothelial cell movement, forming new blood vessel tubes, and ensuring the stability of these newly formed tubes by mesenchymal cells. Activation of the TEK receptor triggers the release of chemical signals that promote communication between endothelial cells and smooth muscle cells, creating new blood vessels and maintaining the structure and integrity of these vessels. In blue rubber bleb nevus syndrome, the TEK receptor is constantly active due to somatic activating mutations, resulting in uncontrolled angiogenesis (Morris et al., 2005; Eklund and Olsen, 2006). This gene has also been implicated in spontaneous and familial venous malformations (VMs), with the *T1105N-T1106P* mutation recurrent in BRBNS. Unlike common unifocal VMs, multifocal malformations are associated with two somatic activating mutations on the same allele, double cis mutations (Soblet et al., 2017). This new understanding of the genetic basis of BRBNS and its relationship to VMs holds promise for improved diagnosis and targeted treatments for affected individuals. In most cases, germline mutations are undetected, while somatic mutations are only found in the affected tissues.

By sequencing the case’s peripheral blood, we discovered the occurrence of germline mutations. The findings are consistent with previous research, and we have identified mutations in the *TEK* gene in two cases. Additionally, our investigation revealed *MMP9, NOTCH3, PRSS1, PDGFRA, CCM2, TSC2*, and *TNFAIP6* alterations in two cases, suggesting a potential contribution to BRBNS. Du et al. demonstrated that the initiation of angiogenesis relies on the essential role of *MMP9* activity, increasing *VEGF* availability (Du et al., 2008). In addition, *CCM2* and *NOTCH3* were reported to be associated with vascular malformations and multiple cerebral cavernous malformations (Snellings et al., 2022; Li et al., 2023). Rare diseases often occur sporadically, with the parents of affected individuals showing normal phenotypes, while the symptoms only appear in the patients themselves. Therefore, rare diseases may occur due to *de novo* mutations. *De novo* mutations refer to postzygotic mutations, including *de novo* germline mutations and somatic cell mutations. When *de novo* mutations occur, the mutated genes should be normal in the patient’s parents. Additionally, the father and mother each may carry one variant allele, leading to compound mutations in the offspring and causing the disease; secondly, the father or mother of the patient may have a variant that does not manifest outwardly, but the patient can express the mutation after inheriting it genetically. In some families, a parent who appears phenotypically normal may have multiple children affected by a penetrant, autosomal dominant, or X-linked disorder. This can be explained by germline mosaicism, where some of the parents’ germ cells carry the harmful allele, which can be passed on to their offspring (Thorpe et al., 2020).

We further performed protein interaction network and enrichment analysis to study these genes' molecular mechanisms in BRBNS. GO and KEGG enrichment analysis showed that *MMP9, NOTCH3, PRSS1, PDGFRA, CCM2, TSC2,* and *TNFAIP6* correlated with vasculature development. Significantly, *TEK*-related genes were related to positive regulation of angiogenesis, positive regulation of vasculature development, and regulation of vasculature development. The activation of *TIE2* and PI3K/AKT pathways independent of ligands is a common feature across all vascular malformations, including BRBN and sporadically occurring VM-causing mutations (Uebelhoer et al., 2013; Nätynki et al., 2015). Limaye et al. have revealed that an additional 20% of unifocal VMs are caused by somatic mutations in *PIK3CA*, which encodes the class I p110a catalytic subunit of *PI3K*, leading to activation of the PI3K/AKT pathway. Clinically, VMs caused by somatic *TIE2* and *PIK3CA* mutations are almost indistinguishable, highlighting the significance of the PI3K/AKT pathway in lesion development downstream of *TIE2* (Limaye et al., 2015). Therefore, cases with these mutations could benefit from PI3K/AKT inhibition, as demonstrated in vascular tumors by studies (Yuksekkaya et al., 2012; Ferrés-Ramis et al., 2015). Our research findings are in line with previous studies. According to the KEGG enrichment analysis results, *MMP9, NOTCH3, PRSS1, PDGFRA, CCM2, TSC2,* and *TNFAIP6* genes are involved in the PI3K-AKT signaling pathway. This indicates that these genes might be crucial in regulating cellular processes associated with the PI3K-AKT pathway, further reinforcing their significance in this biological context.

No standardized treatment is available for Blue Rubber Bleb Nevus Syndrome (BRBNS). Blue rubber bleb nevus syndrome management primarily focuses on treating symptoms and monitoring gastrointestinal lesions to prevent severe bleeding. Cases may receive iron replacement or transfusions if they develop iron deficiency anemia from gastrointestinal bleeding (Agnese et al., 2010). Skin lesions associated with BRBNS do not necessitate specific treatment, and options such as laser photocoagulation, sclerotherapy, or surgical resection are only considered for aesthetic or functional purposes. Treatment options for gastrointestinal (GI) vascular malformations include endoscopic sclerotherapy, band ligation, or laser photocoagulation. While surgical resection may be applicable for localized lesions, GI bleeding, intestinal ischemia, intussusception, and other complications, its efficacy remains controversial due to postoperative lesion recurrence (Arena et al., 2015). Drug therapy options encompass glucocorticoids, interferon-alpha, vincristine, and octreotide, all of which contribute to stabilizing the prognosis of BRBNS and reducing GI bleeding by inhibiting vascular endothelial cell production and proliferation (Aihara et al., 1991; Boente et al., 1999; Garzon et al., 2007). Additionally, it has been shown that the mTOR inhibitor rapamycin (sirolimus) effectively reduces *AKT* phosphorylation, thereby controlling lesion expansion in mice and improving disease outcomes in cases with *PIK3CA* and *TEK*-mediated VMs (Boscolo et al., 2015). Case #1 showed significant improvement in symptoms after treatment with sirolimus during follow-up visits.

These results offer significant insights into the genetic factors linked with BRBNS and could facilitate better comprehension of the underlying mechanisms. Our findings underscore the importance of exploring these genetic alterations in the context of BRBNS, which may have implications for developing targeted therapeutic approaches. Further research is needed to determine the specific roles of these genetic variations in BRBNS pathogenesis and their potential as intervention targets.

## Data Availability

The raw sequencing data generated in this study have been deposited in the NCBI Sequence Read Archive (SRA) under BioProject accession number PRJNA1272012. All data are publicly accessible via the following URL: https://www.ncbi.nlm.nih.gov/bioproject/PRJNA1272012.
